# *IRF4*-Rearranged Large B-Cell Lymphoma on Waldeyer’s Ring: A Case Report

**DOI:** 10.4274/tjh.galenos.2020.2020.0086

**Published:** 2020-11-19

**Authors:** Deram Büyüktaş, Serdar Örnek, Fatma Tokat, Tülay Tecimer, Burhan Ferhanoğlu

**Affiliations:** 1 Koç University Faculty of Medicine, Department of Hematology, İstanbul, Turkey; 2 American Hospital, Clinic of Hematology, İstanbul, Turkey; 3 Acıbadem University Faculty of Medicine, Department of Pathology, İstanbul, Turkey; 4 Acıbadem Health Group, Pathology Laboratory, İstanbul, Turkey

**Keywords:** Large B- cell lymphoma, Waldeyer’s ring, IRF4, MUM1

## To the Editor,

Large B-cell lymphoma (LBCL) with *IRF4* rearrangement is a newly recognized and rare entity. LBCL is characterized by co-expression of *MUM1* and *BCL6*. It has been associated with young age and a favorable outcome. Most patients present with predominantly Waldeyer’s ring or neck or head lymph node involvement [1,2,3]. Here, we present a case of LBCL with *IRF4* rearrangement in an older male.

**Figure 1:**
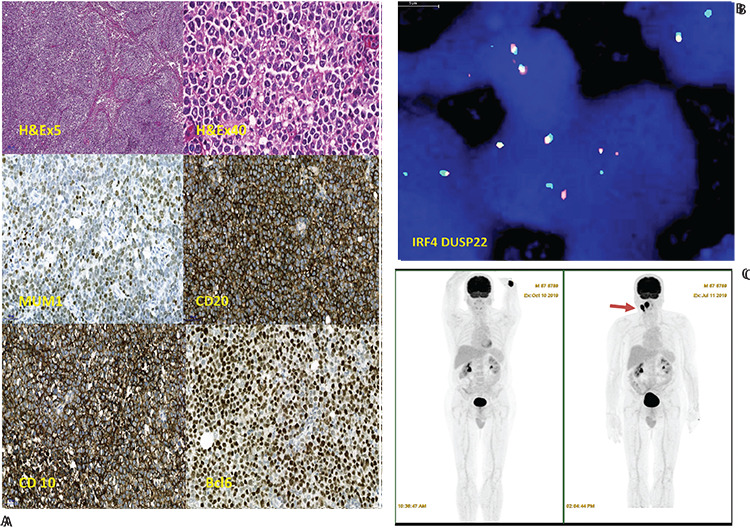
Large B-cell lymphoma with *IFR4* rearrangement. A) Follicular and diffuse pattern, H&E, original magnification 5x. Medium to large-sized neoplastic cells with vesicular chromatin and 2-3 nucleoli, H&E, original magnification 40x. Large cells with positive expression of *MUM1, CD20, CD10*, and *bcl6*, original magnification 20x. B) FISH analysis demonstrated a positive *IRF4* translocation. C) Comparative PET-CT images (before and after treatment).

A 67-year-old man was admitted to the hospital with sore throat, dysphagia, and a lump in the right cervical region. Physical examination showed that the right palatine tonsil was enlarged and there was 2 cm of cervical lymphadenopathy unilaterally. After one week of antibiotic therapy, the lymphadenopathy still persisted. The patient underwent an excisional lymph node biopsy, which was consistent with LBCL of germinal center type, with a mainly follicular and focally diffuse pattern. The immunohistochemistry panel was positive for CD20 (clone L26; Scytek), CD10 (clone 56C6; Biocare), bcl-6 (clone LN22; Biocare Medical), and MUM1 (clone BC5; Biocare Medical), while bcl-2 (clone 124; Scytek) and myc (clone MYC; Biocare) were negative (Figure 1A). Different from follicular lymphoma and diffuse large B-cell lymphoma, the neoplasm was mainly composed of centroblast-like large cells with germinal center phenotype and strong co-expression of *MUM1*. Morphologic and immunohistochemical findings reminded us of the possibility of *IRF4*-rearranged LBCL. FISH analysis for *IRF4* rearrangement (*IRF4*, *DUSP22* dual-color break-apart probe) was performed and rearrangement was found to be positive (Figure 1B). Based on these findings, LBCL with *IRF4* rearrangement was diagnosed. PET-CT showed increased FDG uptake on the right tonsil with level IIA-III cervical stations (Figure 1C). Clinical staging studies led us to stage I disease and the International Prognostic Index score was calculated as 1. After four cycles of R-CHOP_21_, PET-CT showed complete remission (Figure 1C). No further therapy was indicated, and 3 months after the chemotherapy, no evidence was observed of any recurrence.

Salaverria et al. [1] studied 720 lymphomas and screened a group of 427 cases for *IRF4*. They identified 20 lymphomas with proven *IG*/*IRF4* fusion with a median age of 12 years. The *IRF4*-positive lymphomas mostly presented as limited disease in the head and neck region, especially in Waldeyer’s ring, and were associated with better prognosis [1].

Ramis-Zaldivar et al. [4] studied 20 LBCL-*IRF4* pediatric and young adult cases with a median age of 14 years. Eight patients had nodal involvement of the head and neck region, and 8 had tonsillar involvement. Fifteen patients had complete remission after therapy without evidence of relapse for up to 99 months in follow-up; thus, a very favorable outcome was shown among LBCL-*IRF4* cases [4].

Older age is considered to be an adverse prognostic factor in patients with Waldeyer’s ring non-Hodgkin lymphoma and as an independent risk factor for inferior survival among patients with diffuse large B-cell lymphoma [3,5]. FLYER study results showed that four cycles of CHOP chemotherapy had the same effect among young patients with aggressive B-cell lymphoma who had favorable risk profile and stage I-II disease [6]. Our patient had no other risk factors except his age, and he had *IRF4* gene arrangement; thus, we ended the chemotherapy after the fourth cycle with negative PET-CT results.

In conclusion, *IRF4* gene analysis should be considered in patients of any age with Waldeyer’s ring LBCL with germinal center origin, follicular and/or diffuse pattern, and strong *MUM1* expression on pathological examination. The presence of *IRF4* rearrangement may affect the prognosis of the disease and the duration of chemotherapy.
